# Solid-Phase Synthesis and Evaluation of Glycopeptide Fragments from Rat Epididymal Cysteine-Rich Secretory Protein-1 (Crisp-1) ‡

**DOI:** 10.3390/molecules15096399

**Published:** 2010-09-14

**Authors:** Mian Liu, David W. Hamilton, George Barany

**Affiliations:** 1 Department of Chemistry, University of Minnesota, Minneapolis, MN 55455, USA; 2 Department of Genetics, Cell Biology and Development, University of Minnesota, Minneapolis, MN 55455, USA

**Keywords:** glycopeptides, solid-phase synthesis, cysteine-rich secretory protein (Crisp-1), circular dichroism, T_N_ antigen

## Abstract

Three 18-residue peptides with the sequence Glp-Asp-Thr-Thr-Asp-Glu-Trp-Asp-Arg-Asp-Leu-Glu-Asn-Leu-Ser-Thr-Thr-Lys, taken from the *N*-terminus of the rat epididymal *c*ysteine-*ri*ch *s*ecretory *p*rotein (Crisp-1) that is important in the fertilization process, were prepared by Fmoc solid-phase synthesis using a convergent strategy. These peptides were the parent sequence, plus two possible α-*O*-linked T_N_ antigen-containing glycopeptides with a Thr(α-D-GalNAc) residue in place of either Thr^3^ or Thr^4^. During chain assembly, two deletion peptides [des-Asp^2^ and des-Thr(Ac_3_-α-D-GalNAc)] and one terminated peptide [*N*-acetylated 14-mer] arose, as did several peptides in which aspartimide formation had occurred at each of the four possible positions in the sequence. These by-products totaled ~20% of the desired product; they were recognized by HPLC and ESI-MS and removed during the intermediate purifications. Final products, obtained in 15–21% overall yields, were characterized by HPLC purities and ESI-MS. Circular dichroism (CD) spectra for all three purified peptides, recorded in pure water and in trifluoroethanol−H_2_O (1:1), revealed that the presence of a sugar moiety does not significantly impact the sampled conformations. Future biological evaluation could elucidate the nature and locus of sugar modification of Crisp-1, and provide insight as to why Crisp-1 protein E binds sperm irreversibly, in contrast to protein D that lacks a sugar near the *N*-terminus and only binds sperm loosely.

## 1. Introduction

Fertilization involves interaction between a sperm that has originated in, matured, and progressed through the male reproductive tract, and an egg, derived from the ovary [1]. The incompletely-defined process of sperm maturation occurs primarily in the epididymis and involves addition of proteins, glycoproteins, and other molecules to the sperm surface [2,3,4,5,6]. However, once in the female tract, sperm undergo a process called ‘capacitation,’ which involves loss of (or changes) in some the components that had been added in the male tract [5,7,8,9]. Sperm capacitation is an absolute requirement for fertilization [1], insofar as capacitated sperm (but not uncapacitated sperm) can bind to the egg’s extracellular coating (zona pellucida), undergo exocytosis of the acrosomic vesicle (acrosome reaction), and fuse with the egg plasma membrane, thereby completing the process [1]. 

Two proteins of the *c*ysteine-*ri*ch *s*ecretory *p*rotein-1 (Crisp-1) gene family, designated protein D and protein E, are synthesized and secreted in the rat epididymis [10,11,12]. Protein D binds sperm loosely and is easily removed *in vitro,* whereas the binding of protein E to sperm is essentially irreversible [13]. These proteins were purified to homogeneity and shown to inhibit the acrosome reaction, as well as tyrosine phosphorylation of sperm proteins [14]. 

Structurally, proteins D and E are both approximately 32 kDa, and comprise 226 amino acid residues [15]. They apparently differ only by a single immunological determinant found in the *N*-terminus of the latter [16], raising the question of what that determinant is and how it affects the differential behavior of these two proteins. Since purified proteins D and E are blocked at the *N*-terminus, classical protein sequencing approaches were not possible, but comparative mass spectrometry of tryptic digests gave some information [17]. One complication for such studies is that the first arginine in the sequence, starting from the *N*-terminus, is cleaved in protein D, but not in protein E. Overall, protein E is 203 Daltons larger than protein D [17], a modification consistent with a post-translational glycosylation of the *N*-terminus of protein E that is missing from protein D [17]. The precise nature or locus of the sugar is not known, although the fact [18] that the original antibody that recognized only protein E was raised against a fraction of rat sperm glycoproteins retained on a *Ricinus communis* agglutinin column (eluted with 0.1 M galactose) strongly suggests that the sugar moiety on the protein is *N*-acetylgalactosamine (GalNAc); this supposition drove the present synthesis-based work which was designed to prove the presence of GalNAc and determine its position. General methods and results from solid-phase synthesis of glycopeptides have been reviewed previously [19,20,21]. We report here solid-phase synthesis of an 18-mer peptide fragment from Crisp-1 and two corresponding glycopeptides containing a Thr(α-D-GalNAc) residue in place of either Thr^3^ or Thr^4^.

## 2. Results and Discussion

### 2.1. Solid-phase peptide synthesis

**Scheme 1:**
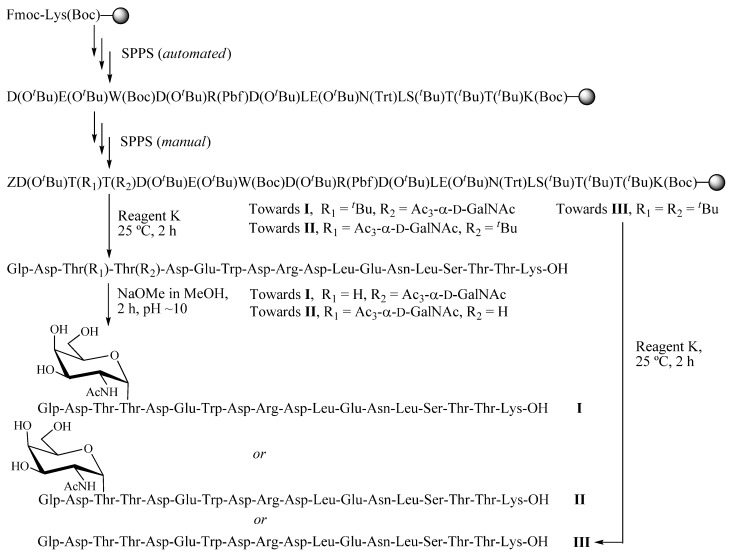
Solid-phase synthesis of glycopeptides **I** and **II**, and peptide **III**, on a CLEAR-acid support.

Three 18-residue peptides were prepared by a combination of automated and manual Fmoc solid-phase synthesis (Scheme 1). The overall sequence is Glp-Asp-Thr-Thr-Asp-Glu-Trp-Asp-Arg-Asp-Leu-Glu-Asn-Leu-Ser-Thr-Thr-Lys, which without further modifications is called III. The glycopeptides containing a Thr(α-D-GalNAc) residue in place of either Thr^4^ or Thr^3^ are termed I and II, respectively. Standard side-chain protecting groups were used. Starting with *C*-terminal Lys(Boc) anchored to a *c*ross-*l*inked *e*thoxylate *a*crylate *r*esin (CLEAR) acid support [22], the first 14 residues were assembled on an automated continuous-flow instrument. Fmoc removal was achieved with piperidine–NMP (1:4) for 30 min, and couplings (four equiv. of Fmoc-amino acids) were mediated by 2-(1*H*-6-chlorobenzotriazole-1-yl)-1,1,3,3-tetramethyluronium hexafluorophosphate (HCTU, 0.45 M in DMF)−HOBt (0.45 M in DMF)−DIEA (1.0 M in NMP) (1:1:2) for 2 h, unless noted otherwise. 

The final four residues were incorporated manually by the same chemistries, using a Mistral multimixer platform to provide vigorous shaking. The Fmoc-Thr(Ac_3_-α-D-GalNAc)-OH building block (1.5 equiv) was coupled for 4 h to compensate for the lower excess, and (for glycopeptide I) results were even better with a second coupling (0.5 equiv). Also, the *N*-terminal pyroglutamyl residue (Glp ≡ Z) was determined through a model peptide synthesis (see “Experimental”) to couple slowly, so for incorporation of this residue, a double coupling protocol was used with a greater excess (8 equiv for each coupling) and an extended time (2 × 4 h). 

Upon completion of solid-phase chain assembly, peptides were cleaved from the support, and amino acid side-chains were simultaneously restored, upon treatment with Reagent K, TFA−thioanisole−1,2-ethanedithiol−phenol−Η_2_Ο (82.5:5:2.5:5:5), at 25 °C for 2 h.

**Figure 1:**
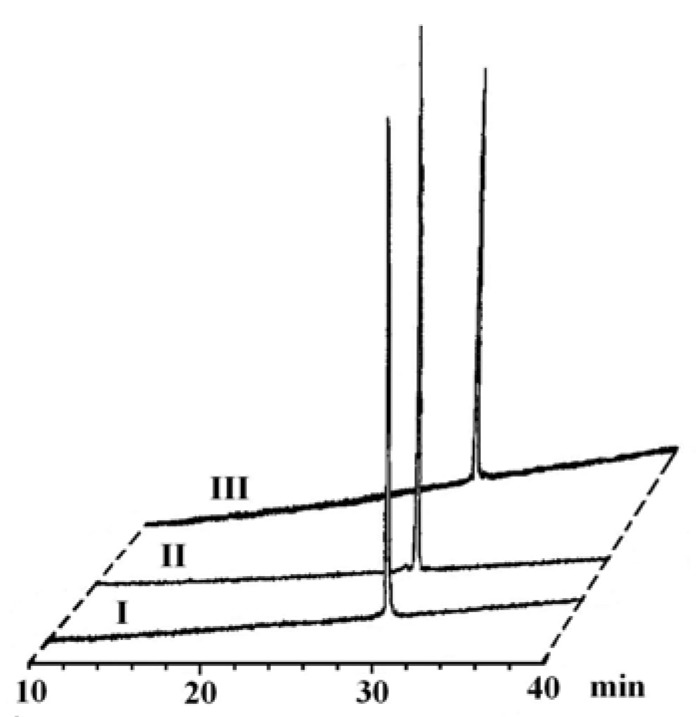
Reversed-phase HPLC of purified glycopeptides **I** and **II**, and peptide **III**.

HPLC purification gave *O*-acetylated glycopeptides which were treated with NaOMe in methanol at pH ~ 10 at 25 °C for 2 h to remove the acetyl groups protecting GalNAc. Final HPLC purification gave respectable yields of glycopeptides I and II, along with the control aglycone III (Figure 1), which were characterized by the appearance of molecular ions with the expected *m*/*z* values upon ESI-MS (Table 1).

Analytical RP-HPLC on a C_18_ column (4.6 × 250 mm), with detection at 220 nm, and elution with 0.1% aq. TFA (buffer A) − 0.1% TFA in CH_3_CN (buffer B), linear gradient from 0 to 40% buffer B, at a flow rate of 1 mL/min for 40 min.

### 2.2. Identification and sources of glycopeptide synthesis by-products 

**Scheme 2:**
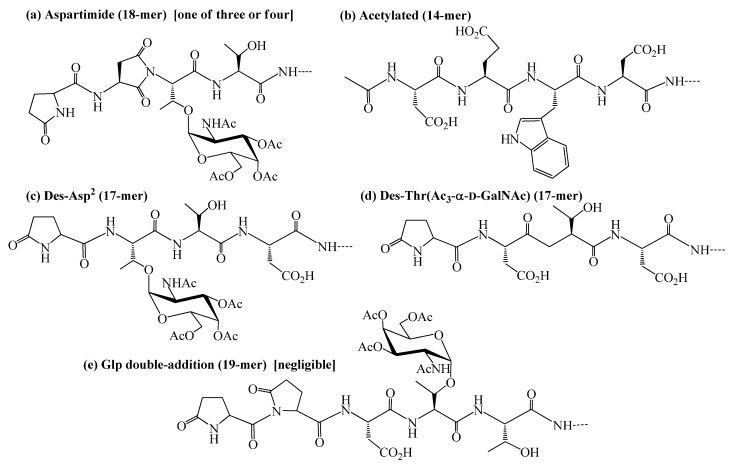
Peptide by-products formed during solid-phase synthesis of glycopeptide **II**.

During the solid-phase protocol outlined in the previous section (Scheme 1), a key purification step was carried out subsequent to removal of all amino acid side-chain protecting groups, but while the sugar remained *O*-acetylated. Several synthetic by-products were revealed at this stage, in amounts of 1 to 6% each (overall ~20%) with respect to the desired product (Scheme 2, Table 2). The major ones are believed to be (**a**) aspartimides, based on ESI-MS data showing loss of H_2_O, and on literature precedent [23,24,25,26,27]. This side reaction seems to have occurred at each of the possible Asp residues, as evidenced by multiple HPLC peaks. Next, a termination peptide termed (**b**) acetylated (14-mer) was noted, which is somewhat difficult to explain other than to note that automated chain assembly gave a 14-mer peptide-resin which was stored for a while before resuming the manual portion of the synthesis. After that, deletion peptides (**c**) des-Asp^2^ (17-mer) and (**d**) des-Thr(Ac_3_-α-D-GalNAc) (17-mer) were observed, reflecting couplings that may have been difficult for steric reasons. Lastly, an unambiguous albeit negligible level of (**e**) Glp double-addition (19-mer) was noted; this corresponds to addition of a second unit of *N*-terminal Glp (introduced without *N*-protection) in an extended coupling cycle for reasons that have already been described.

### 2.3. Circular dichroism spectroscopy

**Figure 2:**
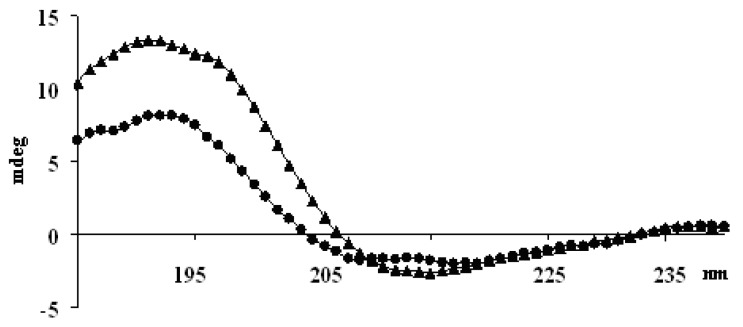
CD spectra of Ac-Thr(α-D-GalNAc)-NH_2_ in pure water (▲; 0.2 mg/mL) and in TFE−H_2_O (1:1; v/v, 0.1 mg/mL) (●). Since the second set of data were obtained after adding one volume of TFE to the pure aqueous solution, the overall concentration of substrate was half of that in the first data set corresponding to pure water.

Far-ultraviolet circular dichroism (CD) spectroscopy is a widely used tool to investigate peptide and protein secondary structure. Below 250 nm, CD spectra of peptides are dominated by the amide chromophore of the chiral peptide backbone [28]. For many studies, trifluoroethanol (TFE) has been used as a structure-inducing cosolvent that can promote and strengthen intramolecular hydrogen bonding and thereby increase the order in peptides that are otherwise unstructured in aqueous solution [29,30,31,32,33]. For GalNAc-containing glycopeptides, the extra amide bond of the acetamido-sugar also needs to be considered as a possible contributor to the CD spectrum. To begin, spectra of Ac-Thr(α-D-GalNAc)-NH_2_ [34] were recorded both in pure water and in TFE−H_2_O (1:1). These showed strong and broad positive bands at ~192 nm, and weak negative bands at ~214 nm, irrespective of the solvent (Figure 2).

**Figure 3:**
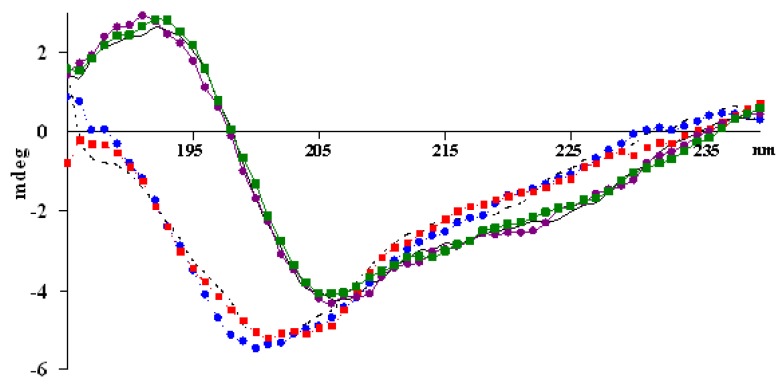
CD spectra of glycopeptides **I** (●) and **II** (■), as well as of peptide **III** (black without symbol) in respectively pure water (0.2 mg/mL; dotted lines) and TFE−H_2_O (1:1; 0.1 mg/mL; solid lines). Again, relative concentrations of the materials in the second set of experiments are half of those in the first set.

CD spectra of aqueous solutions of glycopeptides **I** and **II**, as well as of peptide **III**, are essentially superimposable (Figure 3). They all show a strong negative band at ~201 nm and a shoulder at ~215 nm; these do not fit the CD characteristics of standard structural elements and are interpreted to mean that the structures are disordered. Upon addition of an equal volume of TFE, dramatic changes were noted. Positive bands observed at ~192 nm, along with negative bands at ~206 nm and weak negative band at ~222 nm, are consistent with a mixture of α-helix, β-sheet, and random coil conformational elements [29]. Such solvent-dependent conformational transitions have been described previously and discussed [28]. We conclude, however, that the presence of a sugar moiety on either Thr^3^ or Thr^4^ does not significantly impact the conformations sampled by these Crisp-1 derived peptides. Other investigators have noted, for other glycopeptide systems in the same size range, subtle yet distinct differences induced by the sugar [30,31,32,33].

## 3. Experimental

### 3.1. Materials and instruments 

Materials, solvents, instrumentation, and general methods for solid-phase peptide synthesis were essentially as described in previous publications from one of our laboratories [34,35]. Fmoc-Lys(Boc)-CLEAR-acid resin (initial loading 0.48 mmol/g) was from Peptide International (Louisville, KY, USA) and Fmoc-Thr(Ac_3_-α-D-GalNAc)-OH was from Chem-Impex International (Wood Dale, IL, USA). UV measurements were performed using a Beckman DU 650 spectrophotometer. Far-ultraviolet CD measurements were recorded at 25 °C over the 185−240 nm range, using a Jasco-710 spectro-polarimeter with a cylindrical quartz cell of path length 0.1 cm, and three accumulated scans.

### 3.2. H-Asp(O^*t*^Bu)-Glu(O^*t*^Bu)-Trp(Boc)-Asp(O^*t*^Bu)-Arg(Pbf)-Asp(O^*t*^Bu)-Leu-Glu(O^*t*^Bu)-Asn(Trt)-Leu-Ser(^*t*^Bu)-Thr(^*t*^Bu)-Thr(^*t*^Bu)-Lys(Boc)-CLEAR [‘14-mer peptide-resin’] 

Peptide chain assembly starting with Fmoc-Lys(Boc)-CLEAR-acid resin (0.521 g, 0.48 mmol/g) was carried out in the continuous-flow mode on a Pioneer Peptide Synthesizer (Framingham, MA, USA). Side-chain protection was provided by Boc for Lys and Trp, *^t^*Bu for Ser and Thr, O*^t^*Bu for Asp and Glu, Pbf for Arg, and Trt for Asn. Fmoc removal was achieved with piperidine–NMP (1:4) for 30 min, and couplings (4 equiv of Fmoc-amino acids) were mediated by HCTU (0.45 M in DMF)−HOBt (0.45 M in DMF)−DIEA (1.0 M in NMP) (1:1:2) for 2 h. The title peptide-resin (1.03 g), with its *N*-terminus free, was dried *in vacuo* overnight. An aliquot (~5 mg) was subjected to acidolytic deprotection/cleavage with Reagent K (1 mL) to provide DEWDRDLENLSTTK: analytical RP-HPLC, t_R_ = 28.2 min; ESI-MS: *m/z* 861.4 [M+2H]^2+^, 574.6 [M+3H]^3+^.

### 3.3. Glp-Asp-Thr-Thr(Ac_3_-α-D-GalNAc)-Asp-Glu-Trp-Asp-Arg-Asp-Leu-Glu-Asn-Leu-Ser-Thr-Thr-Lys-OH (*O*-acetylated **I**), Glp-Asp-Thr(Ac_3_-α-D-GalNAc)-Thr-Asp-Glu-Trp-Asp-Arg-Asp-Leu-Glu-Asn-Leu-Ser-Thr-Thr-Lys-OH (*O*-acetylated **II**), and Glp-Asp-Thr-Thr-Asp-Glu-Trp-Asp-Arg-Asp-Leu-Glu-Asn-Leu-Ser-Thr-Thr-Lys-OH (**III**) 

The ‘14-mer peptide-resin’ described above was distributed (150 mg each) to three glass vessels (8.5 mL), each with a porous polypropylene frit and a Teflon-lined screw cap, and the final four residues of glycopeptides **I** and **II**, and of peptide **III**, were added manually. A Mistral multimixer (Lab-Line Instruments, Inc., Melrose Park, IL), modified to contain four horizontal clamps, was used to achieve simultaneous vigorous agitation. All reactions were carried out under dry N_2_ and in the dark, and washings between chemical steps were carried out with NMP and CH_2_Cl_2_. Fmoc removal was achieved with piperidine–NMP (1:4) for 30 min. The couplings of Fmoc-Thr(*^t^*Bu)-OH (4 equiv) or Fmoc-Asp(O*^t^*Bu)-OH (4 equiv) were mediated by HCTU (62 mg, 0.15 mmol, 4 equiv) plus HOBt·H_2_O (23 mg, 0.15 mmol, 4 equiv) plus DIEA (34 μL, 0.18 mmol, 5 equiv) in NMP (2 mL) at 25 °C; a 10-min preactivation period was followed by a 2 h reaction. Fmoc-Thr(Ac_3_-α-D-GalNAc)-OH (38 mg, 56 μmol, 1.5 equiv) was coupled for 4 h in the presence of HCTU (31 mg, 75 μmol, 2 equiv) plus HOBt·H_2_O (12 mg, 75 μmol, 2 equiv) plus DIEA (17 μL, 90 μmol, 2.5 equiv) in NMP (2 mL) at 25 °C; for an improved synthesis of glycopeptide **I**, a second coupling followed with Fmoc-Thr(Ac_3_-α-D-GalNAc)-OH (13 mg, 18.7 μmol, 0.5 equiv), HCTU (11 mg, 26.6 μmol), HOBt·H_2_O (4 mg, 25 μmol), and DIEA (6 μL, 32 μmol) in NMP (1.5 mL). Double couplings of Glp (40 mg, 0.3 mmol, 8 equiv) were mediated by HCTU (124 mg, 0.3 mmol, 8 equiv), HOBt·H_2_O (46 mg, 0.3 mmol, 8 equiv), and DIEA (68 μL, 0.36 mmol, 10 equiv) in NMP (2 mL), at 25 °C. After chain assembly was completed, the peptide-resin was washed with NMP and CH_2_Cl_2_ continuously, transferred to a plastic syringe (10 mL; Torviq, Niles, MI, USA) containing a porous polypropylene frit, and then dried *in vacuo* overnight.

### 3.4. Deprotection/cleavage and purification to provide glycopeptides **I** and **II**, and peptide **III**

*O*-Acetylated glycopeptides, and the aglycone peptide, were cleaved from the corresponding peptide-resins, with concurrent removal of Boc, *^t^*Bu, O*^t^*Bu, Pbf, and Trt side-chain protecting groups, by treatment with reagent K (5 mL) in the dark and under dry N_2_ for 2 h at 25 °C. Each cleaved peptide-resin was then washed extensively with TFA (3 × 1 mL), and the peptides were precipitated by adding cold anhydrous ether (100 mL) to the combined filtrates. The ether-treated materials were kept overnight at 4 °C, and were then collected by centrifugation and washed further with cold ether (2 × 20 mL). The two *O*-acetylated glycopeptides, and the free aglycone peptide, were all purified by semi-preparative RP-PHLC, and the correct fractions were pooled and lyophilized. The lyophilized *O*-acetylated glycopeptides **I** and **II** were then dissolved in methanol (~4 mL/mg) for removal of *O*-acetyl groups. The pH of the methanol solution was adjusted to ~10 (as detected by wet litmus paper) by adding a solution of NaOMe in methanol (0.14 M; 1.2−1.3 mL used). The ensuing *O*-deacetylation reactions, carried out in the dark under N_2_ with mild stirring, were monitored by analytical RP-HPLC and found to be complete after 2 h. Powdered CO_2_ (dry ice) was then added carefully to reach a pH of 6, and the pure glycopeptides were obtained after semi-preparative RP-HPLC followed by lyophilization of the appropriate fractions.

### 3.5. Model peptide Glp-Asp-Trp-Lys

The model peptide was prepared as a preliminary to the main syntheses already described, in order to ascertain the conditions for Glp incorporation, and to confirm that sensitive amino acid side-chains would survive the various conditions used in the overall scheme for glycopeptide synthesis. Chain assembly, starting with Fmoc-Lys(Boc)-CLEAR-acid resin (0.106 g, 0.47 mmol/g), was carried out in the continuous-flow mode (2-h single couplings), and cleavage with reagent K was achieved in the same general manner. The cleaved model peptide-resin was washed with TFA (3 × 0.5 mL), and the combined filtrates were evaporated to provide a residue that was coevaporated with toluene (3 × 10 mL) to remove the last traces of TFA, and then dissolved in water. After lyophilization, the residue was analyzed by ESI-MS and RP-HPLC, and shown to comprise about equal amount of desired Glp-Asp-Trp-Lys, t_R_ = 17.7 min, *m/z* 559.3 [M+H]^+^, and deletion peptide Asp-Trp-Lys, t_R_ = 14.5 min, *m/z* 448.3 [M+H]^+^. This mixture of desired and deletion peptide was dissolved in methanol (2 mL), and the pH was adjusted to 10−11 by adding NaOMe in methanol (0.14 M, 0.3 mL). The two peptides were unchanged over a 7-h period, as monitored by RP-HPLC. 

## 4. Conclusions

Fmoc solid-phase synthesis has been used to successfully prepare three 18-residue peptides, two of which incorporate Thr(α-D-GalNAc), that are related to the epididymal protein Crisp-1 which is important in the fertilization process. During construction of these products, several side reactions were noted by HPLC coupled with ESI-MS analysis; these include dehydration at any of several aspartic acid residues leading to aspartimide formation, incomplete coupling which generated several deletion peptides, and unexpected termination through acetylation at the *N*-terminus of the 14-mer peptide. This last-mentioned peptide corresponded to the convergent intermediate assembled on an automated synthesizer, prior to splitting the peptide-resin into portions for manual introduction of the last four residues. While all of these by-products were eliminated along the way as purification and further chemical steps were carried out, the fact that they were detected suggests alternative strategies to minimize or prevent entirely their occurrence during future syntheses in these families. For example, aspartimide formation can be circumvented either by use of a backbone amide protecting group [24,36] or by incorporation of an appropriate dipeptidyl building block [37]. In other cases, simple double coupling protocols and/or extended coupling times are recommended. The products of this work were evaluated by CD both in water, where they are disordered, and in a mixture of TFE and water, where stable secondary structures were observed but, importantly, these did not depend on the nature of a GalNAc modification or on its position. With the appropriate peptides now available, biological evaluation can proceed.

## Figures and Tables

**Table 1:** Characterization of glycopeptides **I** and **II**, along with peptide **ΙΙΙ**.

Glycopeptides	Molecular Formula	Calcd.	Obsd. (*m*/*z*)^a^	t_R_ (min)^b^	Purity^c^	Yield^d^
*O*-Acetylated **I** Glycopeptide **I**	C_103_H_155_N_25_O_46_ C_97_H_149_N_25_O_43_	2478.06 2352.02	1240.0, 827.0 1177.0, 785.0	33.3 29.4	— >98%	— 18%^e^
*O*-Acetylated **II**	C_103_H_155_N_25_O_46_	2478.06	1240.0, 827.0	32.9	—	—
Glycopeptide **II**	C_97_H_149_N_25_O_43_	2352.02	1177.0, 785.0	29.7	>98%	21%
Peptide **III**	C_89_H_136_N_24_O_38_	2148.94	1075.4, 717.3	30.6	>99%	15%

^a^ ESI-MS: peptide was dissolved in CH_3_CN−H_2_O (1:1), and detected as [peptide+2H]^2+^ and [peptide+3H]^3+^; ^b^ RP-HPLC under conditions of Figure 1 legend; ^c^ Based on analytical RP-HPLC; ^d^ Overall isolated yields, based on initial substitution of resin. Yields were not determined for the *O*-acetylated intermediates; ^e^ A synthesis in which Fmoc-Thr(Ac_3_-α-D-GalNAc)-OH was single coupled gave a 10% yield, whereas the text synthesis used double coupling for this residue.

**Table 2:** By-products to synthesis of *O*-acetylated glycopeptide **II**^a^.

	By-products	Molecular Formula	Calcd.	Obsd. (*m*/*z*)^b^	t_R_ (min)^c^	Amount^d^
**a**	Aspartimide (18-mer)	C_103_H_153_N_25_O_45_	2460.05	1231.0, 821.4	31.5, 31.8, 32.0	8%^e^
**b**	Acetylated (14-mer)	C_74_H_114_N_20_O_30_	1762.8	882.4	30.8	6%
**c**	Des-Asp^2^ (17-mer)	C_99_H_150_N_24_O_43_	2363.03	1182.5, 788.7	33.3	3%
**d**	Des-Thr(Ac_3_-α-D-GalNAc) (17-mer)	C_85_H_129_N_23_O_36_	2047.9	1024.8	26.9	2%
**e**	Glp double-addition (19-mer)	C_108_H_160_N_26_O_48_	2589.09	1295.5, 864.1	34.3	<1%

^a^ Qualitatively similar results were observed for *O*-acetylated glycopeptide **I**; ^b^ ESI-MS: peptide was dissolved in CH_3_CN−H_2_O (1:1), and detected as [peptide+2H]^2+^ and [peptide+3H]^3+^; ^c^ Analytical RP-HPLC under conditions of Figure 1 legend; ^d^ Calculated from analytical RP-HPLC, (by-product)/(*O*-acetylated)**II** × 100%; ^e^ Sum of several resolved HPLC peaks.

## Abbreviations

Ac: acetyl; Boc: *tert*-butyloxycarbonyl; *^t^*Bu: *tert*-butyl; CD: circular dichroism; CLEAR: cross-linked ethoxylate acrylate resin; DIEA: *N,N*-diisopropyl-ethylamine; ESI-MS: electrospray ionization mass spectrometry; Fmoc: 9-fluorenyl-methyloxycarbonyl; Glp: pyroglutamyl residue; HCTU: 2-(1*H*-6-chlorobenzotriazole-1-yl)-1,1,3,3-tetramethyluronium hexafluorophosphate; HOBt: 1-hydroxybenzotriazole; NMP: *N*-methylpyrrolidone; O*^t^*Bu: *tert*-butyloxy; Pbf: 2,2,4,6,7-pentamethyl-dihydrobenzofuran-5-sulfonyl; RP-HPLC: reversed-phase high performance liquid chromatography; SPPS: solid-phase peptide synthesis; TFA: trifluoroacetic acid; TFE: trifluoroethanol; Trt: trityl; Z: Glp residue.
